# Potential benefits of joint hypothetical interventions on diet, lead, and cadmium on mortality in US adults

**DOI:** 10.1186/s12940-022-00905-4

**Published:** 2022-10-05

**Authors:** Nasser Laouali, Tarik Benmarhnia, Youssef Oulhote

**Affiliations:** 1 Department of Biostatistics and Epidemiology, School of Public Health and Health Sciences, University of Massachusetts at Amherst, Amherst, MA USA; 2 Scripps Institution of Oceanography, University of California, San Diego, USA; 3 Centre for Research in Epidemiology and Population Health (CESP): Exposome and Heredity team, Inserm (Institut National de la Santé et de la Recherche Médicale) U1018, Gustave Roussy Institute, 114 rue Edouard Vaillant, 94805 Villejuif Cedex, France

**Keywords:** Metals, Diet, Inflammation, Cardiovascular, Cancer, Mortality, NHANES

## Abstract

**Background:**

Previous studies reported associations between high blood lead levels (BLLs) and urinary cadmium (UCd) concentrations and all-cause and cause-specific mortality. It is hypothesized that these associations are mediated by inflammation; therefore, adherence to an anti-inflammatory diet may mitigate these effects. We sought to estimate the potential effects of joint hypothetical interventions on metals levels and adherence to an anti-inflammatory diet or fruits and vegetables (FV) intake on the expected mortality distributions.

**Methods:**

We used data on 14,311 adults aged ≥ 20 years enrolled in the NHANES-III between 1988 and 1994 and followed up through Dec 31, 2015. We estimated daily FV servings and adherence to the dietary inflammatory index at baseline using 24-hour dietary recalls. Mortality was determined from the National Death Index records. We used the parametric g-formula with pooled logistic regression models to estimate the absolute risk of all-cause, cardiovascular, and cancer mortality under different hypothetical interventions compared to the natural course (no intervention).

**Results:**

Overall, we observed a decreased mortality risk when intervening to lower metals levels or increasing adherence to an anti-inflammatory diet or the daily FV servings. The joint intervention to lower BLLs and UCd and increase the adherence to the anti-inflammatory diet had the strongest impact on cancer mortality risk (risk difference [RD] = -1.50% (-2.52% to -0.62%)) compared to the joint intervention only on metals levels RD= -0.97% (-1.89 to 0.70). The same pattern of associations was observed for the joint intervention to lower both metals and increased daily FV servings and cardiovascular diseases mortality risk.

**Conclusion:**

Higher diet quality may constitute a complementary approach to the interventions to reduce exposures to cadmium and lead to further minimize their effects on mortality. A paradigm shift is required from a pollutant-focused only to a combination with a human-focused approach for primary prevention against these metals.

## Introduction

Lead and cadmium are among the most common environmental and occupational pollutants and represent a significant public health issue. Conventional public health approaches are (1) avoidance of exposure sources and (2) strict regulations based on risk assessment. For example, recently, significant investments have been made by the U.S. Environmental Protection Agency for the removal of lead service lines through regulatory and statutory. As a result of these public health measures, in recent decades, lead and cadmium exposures have declined sharply in the United States [1, 2] which led to reductions in mortality, especially from cardiovascular disease in US adults [3]. We recently showed that interventions to reduce blood lead levels (BLLs) and creatinine-corrected urinary cadmium levels (UCd) from the 95th percentile to the 5th percentile of their distribution in our study population were associated with a lower risk of mortality, especially from cardiovascular and cancer diseases [4]. However, with the lower current levels of metals in the general population, it has become harder to further reduce these levels only through regulations due to the diverse, often ubiquitous, sources of exposure. Thus, a paradigm shift is required from a pollutant-only focused approach to a human-focused approach for health protection against these metals. The human-focused approach can be seen as methods (1) to decrease the body’s burden of pollutants by increasing their excretion from the body and (2) to mitigate early harmful effects of pollutants at cellular levels by the activation of repair and self-recovery systems [5].

While there are many postulated mechanisms through which these metals may act; inflammation and oxidative stress are likely the most important pathways. Lead and cadmium exposures have been positively associated with the systemic inflammation markers alkaline phosphatase, C-reactive protein, and gamma-glutamyl transferase, a known marker of oxidative stress in population studies [6]. On the other side, it is well known that diet is one of the main lifestyle-related factors which can modulate inflammatory processes [7–9].

Observational and experimental studies showed that fruits and vegetables’ antioxidant natural products and bioactive compounds have beneficial effects on several health outcomes by reducing oxidative stress and mitigating inflammation [10, 11]. Interestingly, the role of specific nutrients, food groups, and/or overall dietary quality in the pollutants-induced hazards was recently identified [10, 12–20]. A U.S. cohort study showed that adherence to the Mediterranean diet reduces cardiovascular disease mortality risk related to long-term exposure to air pollutants [14]. In US older adults, we recently reported that adherence to an antioxidant and anti-inflammatory diet may mitigate adverse cognitive effects associated with blood metals including lead and cadmium [13]. Little is known, however, about interventions on a diet that could constitute an efficient and complementary approach to the conventional approaches to further minimize the effects of cadmium and lead exposures. It is unlikely that large-scale and long-term dietary trials will be conducted for the prevention of cadmium and/or lead-induced mortality. Therefore, we used observational data from the third National Health and Nutrition Examination Survey (NHANES) to estimate the potential effects of joint hypothetical interventions on dietary intake of fruits and vegetables (FV) or adherence to an anti-inflammatory diet and metals exposures levels on the expected all-cause and specific causes of mortality distributions.

## Materials and methods

### Design and participants

The NHANES is an ongoing survey conducted by the Centers for Disease Control and Prevention (CDC) that uses a representative sample of non-institutionalized civilians in the US; selected by a complex, multistage, stratified, clustered probability design. Information on participants was collected through interviews and physical examinations. The interview includes background information such as socio-demographic, dietary, and health-related questions. The examination component consists of medical and physiological measurements, as well as laboratory tests. The National Center for Health Statistics Ethics Review Board approved all NHANES protocols, and all survey participants completed a consent form. The detailed protocol on NHANES methodology and data collection are available at https://www.cdc.gov/nchs/nhanes/index.htm. For this study, we included adults aged ≥ 20 years enrolled in the NHANES-III between 1988 and 1994, with data on blood lead and urinary cadmium concentrations (n = 16,040). Then we excluded participants with missing data on mortality and covariates (n = 1,729). The final study population included 14,311.

### Measurements of blood lead and urinary cadmium

Blood and urine samples were collected during the medical examination. The laboratory methods for the processing of these samples are described in detail elsewhere [21]. Briefly, the blood and urine specimens were frozen (− 30 °C and − 20 °C; respectively), stored, and shipped for analysis to the Division of Laboratory Sciences, National Center for Environmental Health at the Centers for Disease Control and Prevention in Atlanta, Georgia (USA). Lead (µg/dL) concentration was measured in whole blood using inductively coupled plasma mass spectrometry. Urinary cadmium was measured by graphite furnace atomic absorption with Zeeman background correction using the CDC and Prevention modification [21] of the method proposed by Pruszkowska et al. [22]. Specimens were analyzed in duplicate, and the average of the two measurements was reported. The detection limits (LOD) were 1.0 µg/dL (0.048 µmol/L) and 0.03 µg/L for BLLs and UCd; respectively. For study participants (n = 1217; 8.5%) who had BLLs below the LOD, a value of $\frac{\text{L}\text{O}\text{D}}{\sqrt{2}}$(0.7 µg/dL) was imputed. Urinary creatinine measured using the Jaffe reaction with a Beckman Synchron AS/ASTRA Clinical Analyzer (Beckman Instruments, Inc., Brea, CA), was used to account for between-participant differences in urine dilution.

### Mortality data

A full description of mortality linkage methods is available from the National Center for Health Statistics (NCHS) [23]. Briefly, the de-identified and anonymized data of the NHANES III participants were linked to National Death Index mortality files based on 12 identifiers for each participant (e.g., Social Security number, sex, and date of birth) with a probabilistic matching algorithm to determine mortality status. The NCHS public-use linked mortality file provides mortality follow-up data from the date of NHANES III survey participation until December 31, 2015 (1988–2015). Participants with no matched death record at this date were assumed to be alive during the entire follow-up period. In a validation study using mortality-linked data from the first NHANES study (NHANES-I; 1971–75), 96% of deceased participants and 99% of those still alive were classified correctly [24]. The underlying cause of death was recorded in the public-use linked mortality files using the following ICD-10 codes: cardiovascular diseases including heart diseases (I00-I09, I11, I13, I20-I51) and cerebrovascular diseases (I60-I69) and malignant neoplasms (C00-C97).

### Dietary inflammatory index and daily fruit and vegetable servings

Dietary intake was collected using 24-hour dietary recalls. The 24-hour recall interview was conducted at a Mobile Examination Center using a standard set of measuring guides to assist in estimating portion sizes. The main fruit and vegetable variable used in these analyses was the seven-digit food coding scheme developed by the United States Department of Agriculture for NHANES III, which categorized items by food group and subgroup. If the main ingredient of a mixed dish was a fruit or vegetable, the item was categorized according to the main fruit or vegetable. Sweets containing fruits where the fruit was not the main ingredient were excluded from the analysis. Serving sizes for each recorded fruit and vegetable were determined using serving size estimations from USDA/U.S. Department of Health and Human Services dietary guidelines. The adapted Dietary Inflammatory Index (DII) was derived from the mean daily intakes of foods/beverages, energy, and nutrients of the 24-hour dietary recalls. We constructed the adapted DII to measure the inflammatory potential of the diet [25]. Briefly, we used the DII index proposed by Woudenberg et al. [26] in combination with the updated dietary components inflammatory weights designed by Shivappa et al. [27] instead of the weights proposed by Cavicchia et al. [28]. These updated inflammatory weights, based on three additional years of published data (2008–2010, inclusive), resulted in a doubling of the total number of articles scored. The DII was based on a nutritional rationale: first, the inflammatory weights of dietary components are multiplied by the standardized energy-adjusted intake, which acts to reduce between-person variation; second, the intakes of all components are standardized; third, the DII calculation did not include alcoholic beverages such as beer, wine, and liquor, total fat, and energy, to avoid overestimation of the inflammatory effects of ethanol, fat, and energy. A total of 24 of the 35 possible dietary components were used for the DII calculation (carbohydrates, proteins, alcohol, fibers, cholesterol, saturated fatty acids, monounsaturated fatty acids, omega 3, omega 6, niacin, thiamin, riboflavin, vitamin B6, vitamin B12, iron, magnesium, zinc, vitamin A, vitamin C, vitamin D, vitamin E, folic acid, beta carotene, and caffeine). A positive DII score indicates an anti-inflammatory diet and negative values correspond to a pro-inflammatory diet.

### Covariates

Baseline covariates were collected when individuals participated in a household interview and demographic information—including sex (male/female), age (continuous; years), ethnicity (Mexican-American, other Hispanic, not Hispanic), income to poverty ratio (categorized in tertiles), the number of years of education attended and completed (continuous; years), area of residence (metro and nonmetro counties), and smoking status (current, former and never) was obtained. Information on body-mass index ([BMI] continuous; kg/m^2^), and physical activity (None, 1 to 14 times, 15 or more times; per month) was obtained during the medical examination. Final models were adjusted for age, sex, ethnicity, income to poverty ratio, educational level, area of residence, smoking status, BMI, and physical activity. Metals concentrations were mutually adjusted. The selection of potential confounders was done between what was available in NHANES using a priori knowledge ensuring adjustment only for confounders and avoiding over-adjustment for potential mediators, colliders, or instrumental variables.

### Hypothetical interventions

For each of the following interventions, we used the parametric g-formula (see details below) to estimate the 27-year mortality risk. There are different policy levers to intervene to reduce or increase metals exposure levels and adherence to an anti-inflammatory diet. Regardless of the policy or action considered, all participants were considered to follow the prescribed intervention beginning at the start of follow-up.

1. Lower BLLs and UCd to the 25th percentile for each metal separately.
2. Lower BLLs and UCd to the 25th percentile for both metals.
3. Increase the dietary inflammatory index to a level corresponding to a more anti-inflammatory (e.g. 75th percentile), or increase the fruit and vegetable daily servings to the number of 5 servings/day as recommended by many nutritional guidelines.
4. Best case scenario: Combining interventions 2 and 3; lower BLLs and UCd and increase the dietary inflammatory index or fruit and vegetable daily servings.
5. Worst case scenario: Increase BLLs and UCd to a level corresponding to the 75th percentile for each metal and lower the DII score to the 25th percentile value of the observed distribution (corresponding to an adherence to a pro-inflammatory diet) or fruit and vegetable daily servings (e.g. number of daily servings < 2).

### Statistical analysis

First, we describe the overall sample BLLs and UCd using geometric means and geometric standard errors (SE) by population characteristics. BLLs and UCd were log_2_-transformed to limit the influence of outliers. We used the parametric g-computation to estimate risk ratios (RR) and risk differences (RD) of all-cause and specific causes of mortality under the above-described hypothetical interventions. In recent years, there have been substantial advances in the application of these methods, and have been used to evaluate hypothetical interventions on sources of lead exposure on BLLs [29]. The parametric g-formula is a generalization of the standardization method, proposed by Robins, [30, 31] and allows for flexibly simulating and estimating the effect of any form of hypothetical intervention. A more detailed discussion of this method is presented elsewhere [32, 33]. Briefly, we first fitted a pooled logistic regression model conditional on covariates and follow-up time, after arranging the data into a person-time structure. We included interaction terms between metal concentrations and dietary exposures in the outcome model. The discrete-time hazards of all-cause and specific causes of mortality for each two-fold increase in the baseline dietary exposure and metals concentrations (log_2_-transformed to reduce skewness) were then estimated. This estimated risk was used to predict the mortality risk under the hypothetical interventions described above. We then compared the estimated risk of 27-year mortality had all participants followed the prescribed interventions with the estimated risk of mortality under (1) no intervention (natural course) and (2) the worst case scenario (Scenario 5).

All analyses were weighted by the provided sample weights to account for the unequal probabilities of inclusion and response rates. We estimated 95% confidence intervals (CIs) of the RR and the RD using non-parametric bootstrap (M = 200) and used the 2.5th and 97.5th percentiles as the lower and upper confidence interval limits, respectively.

Statistical analyses were performed using R version 4.0.4 and the Statistical Analysis System software, version 9.4 (SAS Institute, Cary, NC, USA).

## Results

A total of 14,311 participants were included (mean age 48.0 ± 18.1) for this analysis. BLLs ranged from 0.70 µg/dL to 56.0 µg/dL, with a geometric mean (GM) of 2.97 µg/dL (geometric standard error [GSE] = 0.02). BLLs were higher in older, male, and current and former smoker participants and those who are in the categories of less healthy dietary indices and low poverty to income ratio (Table 1). The urinary creatinine corrected cadmium concentrations (UCd) ranged from 0.002 µg/g to 23.35 µg/g, with a GM of 0.36 µg/g (GSE = 0.003). Participants who had higher UCd were older, and more likely to be female, to be current and former smokers, and to be in the categories of less physical activity. There were no major differences in other participant characteristics (Table 1).

**Table 1:** Blood lead and urinary cadmium geometric means and standard errors by study population baseline characteristics

		Blood lead (µg/dL)	Urinary cadmium (µg/g)
	**N (%)**	**GM (GSE)**	**GM (GSE)**
**Age classes (years)**			
Quartile 1 (< 33)	3,833 (26.78)	2.45 (0.3)	0.22 (0.0)
Quartile 2 (34–45)	3,516 (24.57)	2.65 (0.3)	0.31 (0.1)
Quartile 3 (4663)	3,532 (24.68)	3.29 (0.0)	0.46 (0.0)
Quartile 4(**≥** 64)	3,648 (25.49)	3.69 (0.0)	0.62 (0.0)
**Sex**			
Women	7,570 (52.90)	2.26 (0.0)	0.42 (0.0)
Men	6,741 (47.10)	4.03 (0.0)	0.31 (0.0)
**Ethnicity**			
Mexican-American	3,891 (27.19)	2.99 (0.0)	0.31 (0.0)
Other Hispanic	369 (2.58)	2.88 (0.1)	0.32 (0.0)
Not Hispanic	10,051 (70.23)	2.96 (0.0)	0.39 (0.0)
**Smoking status**			
Never smoker	6,807 (47.7)	2.37 (0.0)	0.27 (0.0)
Current smoker	4,477 (31.28)	3.86 (0.0)	0.48 (0.0)
Former smoker	3,027 (21.15)	3.32 (0.0)	0.46 (0.0)
**Body-mass index**			
Normal weight (< 25·0 kg/m^2^)	5,707 (39.88)	2.91 (0.0)	0.34 (0.0)
Overweight (25·0–29·9 kg/m^2^)	4,855 (33.92)	3.18 (0.0)	0.38 (0.0)
Obese (≥ 30·0 kg/m^2^)	3,749 (26.20)	2.80 (0.0)	0.38 (0.0)
**Physical activity (per month)**			
None	2,939 (20.54)	3.14 (0.0)	0.44 (0.0)
One to 14 times	5,099 (35.63)	2.89 (0.0)	0.37 (0.0)
15 or more times	6,273 (43.83)	2.95 (0.0)	0.32 (0.0)
**Poverty to income ratio tertile**			
First	4,776 (33.37)	3.21 (0.0)	0.38 (0.0)
Second	4,765 (33.30)	2.85 (0.0)	0.36 (0.0)
Third	4,770 (33.33)	2.85 (0.0)	0.35 (0.0)
**Alcohol intake (drinks per month)**			
Four or fewer	9,940 (69.46)	2.72 (0.0)	0.38 (0.0)
More than four	4,371 (30.54)	3.63 (0.0)	0.33 (0.0)
**Healthy eating index tertile**			
First	4,765 (33.30)	3.23 (0.0)	0.36 (0.00)
Second	4,774 (33.36)	2.91 (0.0)	0.35 (0.0)
Third	4,772 (33.34)	2.78 (0.0)	0.38 (0.0)
**Adapted dietary inflammatory index tertile**			
First	4,770 (0.33)	2.79 (0.0)	0.34 (0.0)
Second	4,771 (0.33)	2.95 (0.0)	0.35 (0.0)
Third	4,770 (0.33)	3.17 (0.0)	0.40 (0.0)

During a median follow-up of 22.5 years (IQR 16.3–24.7), 5,167 (36%) participants died with 1,550 (30%) and 1,135 (22%) attributable to cardiovascular disease and cancer, respectively. Table 2 presents the results of the interventions on metals concentrations and the DII. Comparing the estimated 27-year risk under each of the single interventions with the estimated risk under no intervention (natural course), lowering lead concentrations to the 25th percentile [RR: 0.59 (95% CI 0.44 to 0.81)], lowering cadmium concentrations to the 25th percentile [RR: 0.56 (95% CI 0.4 to 0.8)] and increasing DII to a more anti-inflammatory diet [RR: 0.96 (95% CI 0.93 to 0.99)] were all associated with all-cause mortality risk. The joint intervention on metals alone or metals and DII had the strongest impact on all-cause mortality risk with a RR of 0.35 (0.22 to 0.64) and 0.96 0.32 (0.20 to 0.61); respectively. The corresponding RD of these joint intervention on metals alone or metals and DII were − 5.4% (-7.3% to -2.6%)] and − 5.5% (-7.5% to -3.1%); respectively.

When comparing the best-case scenario (lower BLLs and UCd and increase DII score to correspond to an anti-inflammatory diet) to the worst-case scenario (increase BLLs and UCd and lower the DII score to correspond to a pro-inflammatory diet), the RD for all-cause mortality was − 11.5% (-17.8 to -7.2). The same pattern of associations was observed for cancer and cardiovascular disease mortality, except for the single intervention on UCd that was not associated with cancer and cardiovascular disease mortality (Table 2).

**Table 2:** The adjusted risk of all-cause and specific-cause mortality under individual and joint interventions on BLLs, UCd, and DII, using parametric g-formula with pooled logistic regression models^a^

	All-cause mortality			Cancer mortality			Cardiovascular diseases mortality	
**Interventions**	Adjusted risk ratio (95% CI)	Adjusted risk difference (95% CI)		Adjusted risk ratio (95% CI)	Adjusted risk difference (95% CI)		Adjusted risk ratio (95% CI)	Adjusted risk difference (95% CI)
**No intervention** **(natural course)**	1	0		1	0		1	0
Lower BLLs = 5th percentile	**0.59 (0.44 to 0.81)**	**-3.32% (-5.02 to -1.43)**		**0.59 (0.31 to 1.03)**	**-0.79% (-1.39 to 0.03)**		0.63 (0.38 to 1.17)	-0.97% (-1.87 to 0.49)
Lower UCd = 5th percentile	**0.56 (0.41 to 0.79)**	**-3.56% (-5.29 to -1.67)**		0.92 (0.38 to 1.48)	-0.16% (-1.16 to 0.87)		1.09 (0.61 to 1.56)	+ 0.25% (-1.01 to 1.57)
Lower both BLLs and UCd to 5th percentile	**0.35 (0.22 to 0.64)**	**-5.37% (-7.28 to -2.64)**		0.49 (0.12 to 1.39)	-0.97% (-1.89 to 0.70)		**0.43 (0.22 to 0.83)**	**-1.55% (-2.69 to -0.42)**
Anti-inflammatory diet = 75th percentile DII value	**0.96 (0.93 to 0.99)**	**-0.32% (-0.67 to -0.12)**		**0.90 (0.72 to 0.98)**	**-0.18% (-0.65 to -0.04)**		**0.93 (0.83 to 0.99)**	**-0.19% (-0.46 to -0.02)**
Lower both BLLs and UCd and anti-inflammatory diet	**0.32 (0.20 to 0.61)**	**-5.47% (-7.48 to -3.10)**		**0.19 (0.05 to 0.67)**	**-1.50% (-2.52 to -0.62)**		**0.33 (0.09 to 0.70)**	**-1.80% (-2.83 to -0.77)**
Higher BLLs and UCd = 95th percentile and pro-inflammatory diet (25th percentile DII)	**1.72 (1.37 to 2.21)**	**+ 5.87% (2.71 to 10.53)**		1.39 (0.82 to 2.75)	+ 0.79% (-0.43 to 3.51)		1.22 (0.74 to 1.85)	+ 0.59% (-0.77 to 2.58)
**Higher lead and cadmium levels and** **pro-inflammatory diet**	1	0		1	0		1	0
Lower both BLLs and UCd and anti-inflammatory diet	**0.19 (0.10 to 0.37)**	**-11.50% (-17.81 to -7.17)**		**0.15 (0.04 to 0.55)**	**-2.34% (-4.43 to -0.73)**		**0.26 (0.07 to 0.64)**	**-2.44% (-4.54 to -0.82)**

^a^200 iterations were performed for bootstrapping the estimated 95% confidence interval

Table 3 presents the results of the interventions on metals concentrations and FV daily servings. Compared to the natural course, the single intervention to set all participants to a 5 FV serving/day was associated with a slightly lower risk of all-cause [RR: 0.97 (95% CI, 0.91 to 1.00)], cancer [RR: 0.95 (95% CI, 0.73 to 1.00)] and cardiovascular diseases mortality [(RR: 0.97 (95% CI, 0.91 to 0.99)]. The joint intervention on metals and daily FV servings was associated with lower all-cause and cardiovascular diseases mortality risk with a RD of -5.47% (-7.71% to -3.41%)] and − 2.05% (-3.24% to -0.99%); respectively. We did not observe any difference in cancer mortality [RD: +0.86% (-0.41 to 2.63)]. When comparing the best-case scenario (lower BLLs and UCd and increase daily FV servings to 5, the minimum recommended by the US dietary guidelines) to the worst-case scenario (increase BLLs and UCd and lower the daily FV servings to correspond to less than 2), the mortality RDs ranged between − 12.64% (-19.16 to -7.73) for all-cause mortality and − 3.13% (-6.17 to -0.52) for cardiovascular diseases mortality (Table 3).

**Table 3:** The adjusted risk of all-cause and specific-cause mortality under individual and joint interventions on BLLs, UCd, and fruits and vegetables daily servings, using parametric g-formula with pooled logistic regression models^a^

	All-cause mortality			Cancer mortality			Cardiovascular diseases mortality	
**Interventions**	Adjusted risk ratio (95% CI)	Adjusted risk difference (95% CI)		Adjusted risk ratio (95% CI)	Adjusted risk difference (95% CI)		Adjusted risk ratio (95% CI)	Adjusted risk difference (95% CI)
**No intervention** **(natural course)**	1	0		1	0		1	0
Higher FV daily servings ≥ 5	**0.97 (0.91 to 1.00)**	**-0.23% (-0.80 to 0.04)**		**0.95 (0.73 to 1.00)**	**-0.12% (-0.56 to -0.01)**		**0.97 (0.91 to 0.99)**	**-0.10% (-0.31 to -0.02)**
Lower both BLLs and UCd and higher FV daily servings	**0.32 (0.13 to 0.62)**	**-5.47% (-7.71 to -3.41)**		0.30 (0.07 to 1.20)	-1.37% (-2.51 to 0.35)		**0.32 (0.12 to 0.69)**	**-2.05% (-3.24 to -0.99)**
Higher BLLs and UCd = 95th percentile and lower FV daily servings < 2	**1.79 (1.28 to 2.49)**	**+ 6.77% (2.38 to 13.94)**		1.44 (0.80 to 2.33)	+ 0.86% (-0.41 to 2.63)		1.34 (0.64 to 2.26)	+ 1.04% (-0.99 to 3.93)
**Higher lead and cadmium levels and** **lower FV daily servings**	1	0		1	0		1	0
Lower both BLLs and UCd and higher FV daily servings	**0.18 (0.08 to 0.36)**	**-12.64% (-19.16 to -7.73)**		**0.21 (0.04 to 0.88)**	**-2.18% (-4.54 to -0.14)**		**0.24 (0.06 to 0.78)**	**-3.13% (-6.17 to -0.52)**

^a^200 iterations were performed for bootstrapping the estimated 95% confidence interval

## Discussion

Using a large nationally representative sample of US adults, we found that joint interventions on lead and cadmium were more beneficial than individual interventions to reduce mortality. Additionally, we found that such synergistic effects were higher for cancer and cardiovascular diseases mortality. Intervening to increase adherence to an anti-inflammatory diet and decrease lead or cadmium exposure levels showed a higher decreased risk of cancer mortality compared with individual interventions on diet and lead or cadmium.

The metals examined here had been previously evaluated for their potential effects on mortality. Two studies reported that chronic low concentrations of lead exposure were associated with premature death, especially from cardiovascular disease [34, 35]. Several epidemiological studies have examined prospectively the associations of cadmium exposure to the risk of all-causes [36–42], cancer [37, 43–45] and cardiovascular disease mortality [37–40, 43]. Most studies have reported a positive association between urinary cadmium concentrations and mortality, except one study for cardiovascular [38], two studies for cancer [37, 43] and all-cause mortality [36, 38]. We recently showed that interventions to reduce BLLs and UCd were associated with a lower risk of deaths from cardiovascular diseases and cancers [4]. We are not aware of any study that has explored this hypothesis to directly compare our results, although some studies have provided evidence that a healthier diet was an effective protective modulator of environmental toxicant-induced inflammation and human disease [13, 14, 16].

Metals exhibit human toxicity and disease through inflammation and oxidative stress. Thus, it is conceivable that nutrients that can contribute to cellular oxidative stress also can exacerbate or amplify environmental toxicity. On the other hand, nutrients that have antioxidant or anti-inflammatory activity could reduce or prevent compromised health or disease induction from environmental pollutants. Multiple studies have investigated the decreased toxicities of environmental pollutants due to bioactive nutrients of fruits and vegetables such as flavonoids, but many of these studies rely on in vitro assays that lack the complexity of a human organismal approach. Importantly, emerging classes of bioactive food components such as polyphenols also have been shown to modulate the pro-inflammatory effects of environmental toxicants. Studies have shown that a wide array of phenolic compounds such as curcumin and quercetin can decrease toxicant-induced oxidative stress and.

inflammation in multiple cell types and tissues [46, 47]. Bioactive food components also have proved to be effective against cadmium and lead toxicity in both human and animal studies [48, 49]. For example, a protective role for flavonoids and other polyphenols has been suggested through the induction of antioxidant enzyme pathways and increased fecal excretion rates [50, 51]. In addition, antioxidants such as vitamin C supplementation in lead-exposed animals significantly reduced blood, liver, and renal lead levels and associated biochemical changes [52]. The main hypothesis is that bioactive food components such as polyphenols and omega-3 polyunsaturated fatty acids may scavenge the reactive oxygen species and free radicals before they can activate pathways in the pathogenesis of diseases and prevent or decrease toxicant-induced inflammation [53].

This study used the NHANES III dataset, a large, national survey whose findings are generalizable to the U.S. adult non-institutionalized population. Although there are strengths to this study including its large sample size and random sampling, the mutual adjustment between lead and cadmium metals, and most importantly, we used a statistical approach that relaxes the proportional hazard assumption (as previously reported, the risk associated with exposure to lead and cadmium vary throughout the follow-up) and most importantly, we considered the dietary intervention on specific food groups, biomarkers of these food groups and an overall dietary scores. There are also important limitations to note. The key limitation is that for lead we relied on blood concentrations; therefore the cumulative chronic or long-term exposure was not accounted for. In addition, covariates data were only available at baseline. Thus, we could not account for time-varying confounding in this study. Another limitation is that we relied on death certificates for the underlying cause of death, but they are imperfect [54]. Most importantly, although the main potential confounding factors were accounted for, there could be residual confounding due to other genetic and/or environmental factors that were either not measured (e.g. proximity to industrial sources that may release other types of pollutants, paint abatement, superfund sites) or measured inadequately, which may have influenced our findings. Finally, because internal dose metrics cannot define correctly the complete history of exposure and duration, the timing that correlates most strongly with the observed health effect is typically unknown or highly uncertain.

This study focused only on hypothetical interventions related to lead and cadmium exposures and dietary intake to simulate what would have been the benefits of their joint interventions. We recommend that future studies explore other interventions based on, for example, lifestyle factors such as physical activity, which may be another complementary approach to the pollutant and diet-based interventions.

## Conclusion

In conclusion, our findings suggest that higher adherence to an anti-inflammatory diet or fruits and vegetable intake may constitute an efficient, and complementary approach to the measures to reduce exposures to cadmium and lead to further minimize their effects on mortality, especially from cancer and cardiovascular diseases. If confirmed, a paradigm shift is required from a pollutant-focused only to a combination with a human-focused approach for primary prevention against these metals.

## Data Availability

A full list of data sets supporting the results in this research article can be found at: https://wwwn.cdc.gov/nchs/nhanes/nhanes3/default.aspx.
